# High-performing farms exploit reproductive potential of high and low prolific sows better than low-performing farms

**DOI:** 10.1186/s40813-018-0091-8

**Published:** 2018-07-16

**Authors:** Satomi Tani, Carlos Piñeiro, Yuzo Koketsu

**Affiliations:** 1 School of Agriculture, Meiji University, Higashi-mita 1-1-1, Tama-ku, Kawasaki, Kanagawa 214-8571 Japan; 2 PigCHAMP pro Europa S.L., c/Santa Catalina 10, 40003 Segovia, Spain

**Keywords:** Farm effect, High-performing farms, High prolific sows, Lifetime performance, Sow potential

## Abstract

**Background:**

Our objective was to examine the impact of farm effects and sow potential on various aspects of sow performance. We examined the interaction between sow prolificacy groups categorized at parity 1 and farm productivity groups for reproductive performance across parities, and lifetime performance. Data included 419,290 service records of 85,096 sows, on 98 Spanish farms, from first-service as gilts to removal, that were served between 2008 and 2013. Farms were categorized into three productivity groups based on the upper and lower 25th percentiles of the farm means of annualized lifetime piglets weaned per sow over the 6 years: high-performing (HP), intermediate-performing (IP), and low-performing (LP) farms. Also, parity 1 sows were categorized into three groups based on the upper and lower 10th percentiles of piglets born alive (PBA) as follows: 15 piglets or more (H-prolific), 8 to 14 piglets, and 7 piglets or fewer (L-prolific). The farm groups represent farm effects, whereas the sow groups represent sow potential. Linear mixed effects models were performed with factorial arrangements and repeated measures.

**Results:**

Mean parity at removal (4.8 ± 0.01) was not associated with three farm productivity groups (*P* = 0.43). However, HP farms had 7.7% higher farrowing rates than LP farms (*P* <  0.05). As a result, H-prolific and L-prolific sows on HP farms had 29.7 and 30.7 fewer non-productive days during lifetime than the respective sows on LP farms (*P* <  0.05). Furthermore, the H-prolific and L-prolific sows on HP farms had 4.9 and 6.2 more annualized piglets weaned than respective H-prolific and L-prolific sows on LP farms (*P* <  0.05), which was achieved by giving birth to 0.8–1.0 and 1.4–1.7 more PBA per litter, respectively, than on HP farms during parities 2–6 (*P* <  0.05). During the first parity, HP farms had 18.8% H-prolific sows compared to 6.2% on LP farms.

**Conclusion:**

Farm effects substantially affected lifetime performance of sows. Higher lifetime productivity of sows on HP farms was achieved by higher farrowing rate, fewer non-productive days, more PBA and more piglets weaned per sow, regardless of prolific category of the sows.

## Background

Prolificacy performance, such as the number of piglets born alive (PBA), appears to differ between individual sows due to the extent of genetic improvement and farm management [1]. Studies in Japan, Europe, and the U.S.A. have shown that the most prolific sows, categorized by PBA at parity 1, produce 0.5–1.8 more PBA from parities 2 to 6 and 1.4–26.0 more lifetime PBA than other sows [2–4]. However, reproductive performance varies between individual sows on a farm, and it is important to maximize the lifetime reproductive performance of all sows in order to decrease production costs and economic wastefulness on the farms [5]. Also, a study of high-performing (HP) farms in the U.S.A., categorized by herd reproductive productivity, showed that they had 9.0% higher farrowing rates, and 0.6 more PBA than ordinary farms [6]. The high productivity of HP farms is attributable to better replacement gilt development [7], better breeding management [8], more advanced technologies [9, 10] and better piglet care during lactation [11, 12]. These studies have shown that HP farms appear to exploit sows’ reproductive potential better than ordinary farms. Therefore, we have hypothesized that high prolific (H-prolific) and low prolific (L-prolific) sows on HP farms perform differently from equivalent sows on intermediate-performing (IP) or low-performing (LP) farms. It is useful for veterinarians and producers to know a quantified association between sows’ potential and farm effects for reproductive performance across parities and lifetime performance of sows. Therefore, the objective of the present study was to examine the interaction between sow prolificacy groups and farm productivity groups for reproductive performance in consecutive parities, and lifetime performance of H-prolific and L-prolific sows. The farm groups represent farm effects, whereas the sow groups represent sow potential.

## Methods

### Studied farms and data selection

A consultancy firm (PigCHAMP pro Europa S.L. Segovia, Spain) has annually requested all client producers to mail their data files since 1998. In 2013, 98 Spanish farms allowed their farm data to be used for research purposes. Our study database included approximately 0.5% of all Spanish pig breeding farms and approximately 4% of all gilts and sows. Spain is one of the major pig producing countries in Europe, with 19,630 breeding farms and 2,568,450 breeding pigs, in December of 2013, accounting for 20% of breeding pigs in the 28 EU countries [13].

The mean (± SEM) size of the studied farms was 699 ± 64.3 sows with a range between 81 and 3222 sows. The study herds increased in size by 14.2% over the 6 years when data were collected. These 98 farms use mechanical or natural ventilation systems in their farrowing, breeding and gestation barns. The lactation and gestation diets were formulated using cereals (barley, wheat and corn) and soybean meal. Also, all the farms use artificial insemination, with double or triple inseminations of sows during an estrous period. Replacement gilts on the 98 farms are either purchased from breeding companies or are home-produced through their internal multiplication programs. These farms’ data were also used for another study to examine risk factors associated with severe repeat-breeder sows [14].

### Study design, data collection and exclusion criteria

The present study was designed as a retrospective cohort study coordinating by-parity service records and subsequent reproductive data in sows, from first-service of gilts to their removal. The data included 554,755 service records of sows served on the 98 farms from January 2008 to June 2013. Data from the PigCHAMP recording system were collected for 99,533 sows entered into the farms between 2008 and 2010. When the data were collected, 4842 (4.8%) of the sows had not yet been removed from the farms, so these records were excluded. Also, lifetime records were excluded if they met any of the following criteria (99th percentile): lifetime non-productive days of 290 days or more (949 sows); lifetime PBA of 130 piglets or more (857 sows), 104 or more lifetime piglets weaned (914 sows), and gilt records of removal at parity 0 (6875 gilts). Additional exclusions were made for no records of gilt age at first-mating (3477 gilts) or records with either less than 160 days (1435 gilts) or more than 400 days (1300 gilts; [15]) when age at first service was analyzed. Parity records of sows in parity 7 or higher were omitted for by-parity reproductive performance analyses (18,264 records), but were included in analysis of lifetime performance. Thus, the studied data for datasets 1 and 2 contained 419,290 first-served records of 85,096 sows on the 98 farms.

Datasets 1 and 2 were created for analyses of by-parity reproductive performance and lifetime performance, respectively. In Dataset 1, service records were regarded as missing records if they met any of the following criteria; more than 26 PBA (1 record), more than 26 piglets weaned (50 records), more than 35 days of weaning-to-first-mating interval (3420 records), and re-service interval of either less than 11 days or more than 150 days (401 records).

### Categories and definitions

Farms were categorized into three groups based on the upper and lower 25th percentiles of the farm means of annualized lifetime piglets weaned per sow: HP farms (> 24.7 piglets), IP farms (24.7 to 21.2 piglets), and LP farms (< 21.2 piglets). Also, sows were categorized into three groups based on the upper and lower 10th percentiles of PBA at parity 1: H-prolific (15 piglets or more), I-prolific (8 to 14 piglets), and L-prolific (7 piglets or fewer) sows.

Lifetime PBA was defined as the sum of the number of PBA in a sow’s lifetime. Annualized lifetime piglets weaned per sow was defined as the lifetime number of weaned piglets divided by the sum of the reproductive herd life days × 365. Reproductive herd life days was defined as the number of days from the date that gilts were first-mated to their removal [16]. Lifetime non-productive days of a sow were defined as the number of days when the sow was neither gestating nor lactating during her reproductive herd life.

### Statistical analysis

All statistical analyses were conducted using SAS version 9.3 (SAS Institute Inc., Cary, NC, U.S.A.). A chi-square test was conducted using SAS software to compare the relative frequencies (%) of sow groups in the different farm productivity groups. Two statistical models were created: Model 1 was applied to Dataset 1 with a 3 × 3 × 6 factorial arrangement design with repeated measures. The analysis was conducted using the three sow groups, three farm groups, six parity groups, and entry years as fixed effects for reproductive performance. Model 1 also examined possible 2- or 3-way interactions. Also, Model 2 was applied to Dataset 2 with a 3 × 3 factorial arrangement, with fixed effects being the three sow groups, three farm groups and entry years. Model 2 also examined possible 2-way interactions.

For continuous outcomes, linear mixed effects models were used to account for the clustering of sows within a farm (MIXED, random statement) or the correlation between repeated measures in the same sow within a farm (MIXED, repeated statement). For binary outcomes, a generalized mixed effects logistic regression model was used with a logit link function in individual parity records (for whether or not a sow farrowed, 1 or 0: farrowing rate). This model was used to account for the clustering of sows within a farm (GLIMMIX, random statement), and the correlation between repeated measures in the same female pig within a farm (GLIMMIX, random_residual_statement). Additionally, if the 3-way interactions between the sow, farm and parity groups were found significant, then we also separately examined 2-way interactions between the farm groups and parity groups for either H-prolific or L-prolific sows. Pairwise multiple comparisons were performed by using the Tukey-Kramer test. All significance levels were set at *P* <  0.05. Also, a random farm effect was included in all the models.

### Intraclass correlation coefficients

The intraclass correlation coefficient (ICC) was calculated by the following equations to assess the variance in the reproductive performance that could be explained by the farms, and also the variance in reproductive performance across parities that could be explained by the sow effect [17],

$$ \mathrm{ICC}\ \left(\mathrm{individual}\ \mathrm{records}\ \mathrm{within}\ \mathrm{the}\ \mathrm{same}\ \mathrm{farm}\ \mathrm{but}\ \mathrm{different}\ \mathrm{sows}\right)\ \mathrm{for}\ \mathrm{continuous}\ \mathrm{outcomes}={\sigma}_v^2/\left({\sigma}_v^2+{\sigma}_{\varepsilon}^2\right), $$

$$ \mathrm{ICC}\ \left(\mathrm{individual}\ \mathrm{parity}\ \mathrm{records}\ \mathrm{within}\ \mathrm{the}\ \mathrm{same}\ \mathrm{sow}\right)\ \mathrm{for}\ \mathrm{continuous}\ \mathrm{outcomes}=\left({\sigma}_v^2+{\sigma}_u^2\right)/\left({\sigma}_v^2+{\sigma}_u^2+{\sigma}_{\varepsilon}^2\right), $$

$$ \mathrm{ICC}\ \left(\mathrm{individual}\ \mathrm{records}\ \mathrm{within}\ \mathrm{the}\ \mathrm{same}\ \mathrm{farm}\ \mathrm{but}\ \mathrm{different}\ \mathrm{sows}\right)\ \mathrm{for}\ \mathrm{binary}\ \mathrm{outcomes}={\sigma}_v^2/\left({\sigma}_v^2+{\pi}^2/3\right), $$

$$ \mathrm{ICC}\ \left(\mathrm{individual}\ \mathrm{parity}\ \mathrm{records}\ \mathrm{within}\ \mathrm{the}\ \mathrm{same}\ \mathrm{sow}\right)\ \mathrm{for}\ \mathrm{binary}\ \mathrm{outcomes}=\left({\sigma}_v^2+{\sigma}_u^2\right)/\left({\sigma}_v^2+{\sigma}_u^2+{\pi}^2/3\right), $$

in which ${\sigma}_v^2$ is the between-farm variance, ${\sigma}_u^2$ is the between-sow variance, and ${\sigma}_{\varepsilon}^2\kern0.5em$or *π*^2^/3 is the assumed variance at the individual record level.

## Results

Descriptive statistics of lifetime performance and by-parity reproductive performance of sows are shown in Table 1. The proportions of H-, I- and L-prolific sows at parities 1 and 6 differed between the three farm groups (*P* <  0.05; Table 2). In parity 1, HP farms had 18.8% H-prolific sows and 9.5% L-prolific sows, whereas LP farms had 6.2% H-prolific sows and 17.6% L-prolific sows. Also, in parity 6 there were 20.9% H-prolific sows and 6.5% L-prolific sows on HP farms, compared with 5.9 and 15.4%, respectively on LP farms.

**Table 1:** Reproductive data for sows on 98 farms

			Range	
	N	Mean ± SEM	Minimum	Maximum
Lifetime performance measurements				
Number of parity at removal	85,096	4.8 ± 0.01	1	11
Gilt age at first-mating, days old^a^	78,884	251.3 ± 0.15	160	400
Lifetime number of piglets born alive	85,096	57.3 ± 0.11	0	129
Lifetime number of piglets weaned	85,096	49.9 ± 0.09	0	103
Annualized lifetime piglets weaned per sow	85,096	23.9 ± 0.02	0	74^c^
Lifetime non-productive days	85,096	84.8 ± 0.17	0	289
Parity performance measurements				
Parity	419,290	2.4 ± 0.01	0	6
Number of piglets born alive^b^	352,457	12.0 ± 0.01	0	26
Number of piglets weaned^b^	352,408	10.5 ± 0.01	0	26
Lactation length, days^b^	348,032	23.5 ± 0.01	14	41
Weaning-to-first-mating interval, days^b^	349,038	5.9 ± 0.01	0	35
Re-service interval, days	37,617	37.5 ± 0.14	11	150

^a^The remaining records (85,096-N) were regarded as missing records

^b^The remaining records (419,290-N) were regarded as missing records

^c^This is a value based on the maximum number of piglets weaned by a sow in parity 1, adjusted to an annualized equivalent (some sows were culled at parity 1, meaning that there are no subsequent data for parity 2 or higher)

**Table 2:** By-parity relative frequencies (%) of farm groups in three sow groups categorized by piglets born alive in parity 1^a^

		Sow groups^b^			Chi-square test
		High prolific	Intermediate prolific	Low prolific	
Farm groups^c^	N^d^	sows, %	sows, %	sows, %	
		Parity 1			
High-performing farms	35,274	18.8	71.7	9.5	
Intermediate-performing farms	37,263	10.1	77.6	12.3	
Low-performing farms	12,559	6.2	76.2	17.6	< 0.01
		Parity 6			
High-performing farms	11,411	20.9	72.6	6.5	
Intermediate-performing farms	12,917	10.2	79.9	9.9	
Low-performing farms	4372	5.9	78.7	15.4	< 0.01

^a^Frequencies within a row add up to 100%

^b^Groups based on the upper and lower 10th percentiles of piglets born alive in parity 1: High (15 piglets or more); Intermediate (8 to 14 piglets) and Low (7 piglets or fewer) prolific sows

^c^Categorized by farm means of the upper and lower 25th percentiles of annualized lifetime piglets weaned per sow over 6 years: High- (> 24.7 pigs); Intermediate- (24.7 to 21.2 piglets) and Low- (< 21.2 piglets) performing farms

^d^N means the number of sows

There were three significant main effects, namely sow groups, farm groups and parity groups with 2-way and 3-way interactions between the groups for both PBA and the number of piglets weaned (*P* <  0.01). Also, there were 2-way interactions between the farm groups and parity groups for PBA and the number of piglets weaned for both H-prolific and L-prolific sows in both dataset models (*P* <  0.01; Appendixes A and B).

Table 3 shows comparisons between the three farm groups for PBA and the number of piglets weaned at subsequent parities by H-prolific and L-prolific sows. The H-prolific sows in all farm groups had more PBA in parity 1 than in parities 2–6 (*P* <  0.05). In contrast, L-prolific sows had more PBA in parities 2–6 than in parity 1 (*P* <  0.05). At parities 2–6, H-prolific and L-prolific sows on HP farms had 0.8–1.1 and 1.4–1.7 more PBA (6–8% and 12–15% more) than the respective sow groups on LP farms (*P* <  0.05). Additionally, the H-prolific and L-prolific sows in all parity groups on HP farms had 1.0–1.6 and 1.4–2.3 more piglets weaned (11–17% and 13–17% more) than the respective sow groups on LP farms (*P* <  0.05).

**Table 3:** Comparisons of reproductive performance of sows during the first parity compared with the subsequent five parities in high, intermediate and low-performing farms of either high prolific or low prolific sows ^1, 2^

		Subsequent parity					
Farm groups^3^	N^4^	1	2	3	4	5	6
High prolific sows^5^							
		Piglets born alive					
High-performing farms	6232	15.8 (0.10)w	13.1 (0.10)az	13.7 (0.10)ax	13.8 (0.10)ax	13.6 (0.10)ax	13.3 (0.11)ay
Intermediate-performing farms	3541	15.8 (0.08)w	12.7 (0.08)abz	13.1 (0.09)bx	13.0 (0.09)bxy	12.8 (0.09)byz	12.7 (0.10)byz
Low-performing farms	732	15.6 (0.14)w	12.3 (0.14)bx	12.7 (0.15)bx	12.8 (0.16)bx	12.5 (0.17)bx	12.3 (0.19)bx
		Piglets weaned					
High-performing farms	6232	11.2 (0.09)aw	11.3 (0.09)aw	11.2 (0.09)aw	11.1 (0.09)ax	10.9 (0.09)ax	10.9 (0.10)ax
Intermediate-performing farms	3541	10.8 (0.07)bw	10.7 (0.07)bwx	10.5 (0.07)bxy	10.4 (0.08)byz	10.4 (0.08)byz	10.2 (0.08)bz
Low-performing farms	732	10.2 (0.12)cw	9.9 (0.12)cw	9.9 (0.12)cw	9.8 (0.13)cw	9.8 (0.13)cwx	9.3 (0.15)cx
Low prolific sows^5^							
		Piglets born alive					
High-performing farms	2924	5.0 (0.10)z	11.8 (0.10)ay	12.5 (0.10)ax	12.8 (0.11)aw	12.9 (0.11)aw	12.7 (0.13)awx
Intermediate-performing farms	4058	5.3 (0.07)y	11.0 (0.07)bx	11.6 (0.08)bw	11.8 (0.08)bw	11.7 (0.08)bw	11.8 (0.09)bw
Low-performing farms	1903	5.1 (0.10)y	10.3 (0.10)cx	11.1 (0.11)cw	11.1 (0.11)cw	11.2 (0.12)bw	11.3 (0.13)bw
		Piglets weaned					
High-performing farms	2924	10.4 (0.11)ax	11.1 (0.11)aw	11.1 (0.11)aw	11.2 (0.11)aw	11.1 (0.12)aw	11.0 (0.13)aw
Intermediate-performing farms	4058	9.4 (0.08)bx	10.3 (0.08)bw	10.3 (0.08)bw	10.2 (0.08)bw	10.2 (0.09)bw	10.2 (0.09)bw
Low-performing farms	1903	8.1 (0.11)cx	9.7 (0.11)cw	9.7 (0.11)cw	9.8 (0.12)bw	9.7 (0.12)bw	9.5 (0.13)cw

^a-c^Different superscripts within a column represent significant differences in means (*P* ≤ 0.03)

^w-z^Different superscripts within a row represent significant differences in means (P ≤ 0.03)

^1^Means and SE were estimated by mixed models

^2^There were no differences between the farm groups in any parity for weaning-to-first-mating interval and farrowing rate (P ≥ 0.05)

^3^Categorized by farm means of the upper and lower 25th percentiles of annualized lifetime piglets weaned per sow over 6 years: high- (> 24.7 piglets); intermediate- (24.7 to 21.2 piglets) and low- (< 21.2 piglets) performing farms

^4^N represents the initial number of sows

^5^Groups based on the upper and lower 10th percentiles of piglets born alive in parity 1: High (15 piglets or more) and Low (7 piglets or fewer) prolific sows

There were three significant main effects for farrowing rate, namely sow groups, farm groups and parity groups, namely sow groups, farm groups and parity groups, as well as a 2-way interaction between sow groups and parity groups (*P* <  0.01; Appendix C). For weaning-to-first-mating interval, there was an association with parity (*P* <  0.05), but not with either sow groups or farm groups (*P* ≥ 0.45). Additionally, there were no 2- or 3-way interactions between these three factors for weaning-to-first-mating interval (*P* ≥ 0.05). Appendix D shows the mean values of reproductive performance in consecutive parities of the H-prolific and L-prolific sows in the three farm productivity groups. With regard to the ICC, the random herd and sow effects explained 1.9–6.0% of the total variance for reproductive performance.

Table 4 shows comparisons between the three factors for farrowing rates and weaning-to-first-mating interval. There were no differences between any of the sow groups or between any of the farm groups for weaning-to-first-mating interval. Regarding farrowing rate, HP farms had 7.7% higher farrowing rates than LP farms, whereas H-prolific sows had 0.7% higher farrowing rates than L-prolific sows (*P* <  0.05). Additionally, Table 5 shows comparisons of farrowing rates between the parity groups for different sow groups. In parities 1 and 2, farrowing rates were 2.7–3.5% higher in H-prolific sows than in L-prolific sows in all the farm groups, but there were no differences between the sow groups for farrowing rates in parities 3 and 6 (*P* ≥ 0.05).

**Table 4:** Comparisons between factors for farrowing rates and for weaning-to-first-mating intervals^f^

	Farrowing rate, %		Weaning-to-first-mating interval, days	
Measurements	N^g^	Mean (± SE)	N	Mean (± SE)
Farm groups^h^				
High-performing farms	181,358	89.3 (0.54)a	145,196	5.9 (0.16)
Intermediate-performing farms	191,554	85.5 (0.50)b	152,492	6.1 (0.11)
Low-performing farms	64,642	81.6 (0.83)c	51,350	6.2 (0.16)
Sow groups^i^				
High prolific sows	58,327	85.8 (0.39)a	46,758	6.0 (0.09)
Intermediate prolific sows	332,716	86.2 (0.35)a	266,291	6.0 (0.08)
Low prolific sows	46,511	85.1 (0.41)b	35,989	6.1 (0.09)
Parity groups				
0	85,096	88.2 (0.34)a	–	–
1	79,267	82.9 (0.45)e	77,447	7.2 (0.08)a
2	70,061	86.4 (0.39)b	69,497	6.1 (0.08)b
3	61,429	86.3 (0.40)bc	61,033	6.0 (0.08)c
4	52,372	86.1 (0.41)bc	52,074	5.8 (0.08)d
5	42,365	85.4 (0.44)cd	42,198	5.7 (0.09)e
6	28,700	84.5 (0.50)d	28,586	5.6 (0.09)e

^a-e^Different superscripts within a column represent significant differences in means (*P* ≤ 0.01)

^f^Means and SE were estimated by mixed models

^g^N represents the number of parity record

^h^Categorized by farm means of the upper and lower 25th percentiles of annualized lifetime piglets weaned per sow over 6 years: High- (> 24.7 piglets); Intermediate- (24.7 to 21.2 piglets) and Low- (< 21.2 piglets) performing farms

^i^Groups based on the upper and lower 10th percentiles of pigs born alive in parity 1: High (15 piglets or more); Intermediate (8 to 14 piglets) and Low (7 piglets or fewer) prolific sows

**Table 5:** Comparisons of farrowing rates (%) between the three sow groups in subsequent parities^1^

		Subsequent parity						
		0	1	2	3	4	5	6
Sow groups^2^	N^3^	Mean (± SE), %						
High prolific sows	11,152	87.9 (0.45)bv	84.3 (0.54)ay	87.3 (0.49)avw	86.7 (0.52)vwx	86.0 (0.57)wxy	84.8 (0.63)xy	83.3 (0.75)y
Intermediate prolific sows	63,824	89.2 (0.31)av	83.5 (0.43)az	87.0 (0.36)aw	86.6 (0.37)wx	86.2 (0.39)x	85.7 (0.41)xy	84.9 (0.45)y
Low prolific sows	10,120	87.4 (0.46)bv	80.8 (0.62)bx	84.6 (0.56)bw	85.6 (0.57)vw	86.1 (0.59)vw	85.6 (0.66)vw	85.3 (0.78)vw

^a, b^Different superscripts within a column represent significant differences in means (*P* <  0.01)

^v-z^Different superscripts within a row represent significant differences in means (*P* <  0.01)

^1^Means and SE were estimated by mixed models

^2^Groups based on the upper and lower 10th percentiles of piglets born alive in parity 1: High (15 piglets or more); Intermediate (8 to 14 piglets); Low (7 piglets or fewer) prolific sows

^3^N represents the initial number of sows

Table 6 shows comparisons of lifetime performance between the three sow groups and three farm groups. There were 2-way interactions between the sow groups and farm groups for lifetime PBA, lifetime piglets weaned and annualized lifetime piglets weaned (*P* <  0.05). Across the farm groups, H-prolific sows had 20.6–25.9 more lifetime PBA (45–58% more) than L-prolific sows, whereas across the sow groups HP farms had 6.1–6.7 more lifetime PBA (7–11% more) than LP farms (*P* <  0.05). In contrast, the differences between HP farms and LP farms for annualized lifetime piglets weaned was greater than the differences between H-prolific sows and L-prolific sows. In detail, across sow groups HP farms had 4.9–6.2 more annualized lifetime piglets weaned (23–34% more) than LP farms. Meanwhile, across farm groups H-prolific sows had 1.3–2.6 more annualized lifetime piglets weaned (5–14% more) than L-prolific sows. The largest difference was between L-prolific sows on HP farms and LP farms. Additionally, across the sow groups, HP farms had 29.7–30.7 fewer lifetime non-productive days (27–30% fewer) than LP farms, whereas across the farm groups H-prolific sows had 5.4–9.0 more lifetime non-productive days (6–12% more) than L-prolific sows.

**Table 6:** Comparisons of reproductive performance between three farm productivity groups and between three sow groups categorized by piglets born alive in parity 1^1^

	Sow groups^2^		
	High prolific sows	Intermediate prolific sows	Low prolific sows
Farm groups^3^	Mean (± SE)	Mean (± SE)	Mean (± SE)
	Number of sows		
High-performing farms	6624	25,318	3332
Intermediate-performing farms	3744	28,944	4575
Low-performing farms	784	9562	2213
	Gilt age at first-mating, days old^4^		
High-performing farms	257.7 (6.05)x	257.3 (6.04)y	256.7 (6.06)y
Intermediate-performing farms	249.7 (4.31)x	248.1 (4.28)y	247.6 (4.30)y
Low-performing farms	259.3 (6.03)x	256.5 (5.93)y	255.8 (5.96)y
	Parity at removal^4^		
High-performing farms	5.1 (0.12)x	5.0 (0.11)x	4.2 (0.12)y
Intermediate-performing farms	5.1 (0.09)x	5.1 (0.08)x	4.6 (0.09)y
Low-performing farms	4.9 (0.14)x	4.9 (0.11)x	4.3 (0.12)y
	Lifetime piglets born alive		
High-performing farms	70.3 (1.40)ax	61.9 (1.36)ay	44.4 (1.45)z
Intermediate-performing farms	66.4 (1.07)abx	59.3 (0.97)aby	45.8 (1.05)z
Low-performing farms	63.6 (1.71)bx	55.8 (1.36)by	41.3 (1.48)z
	Lifetime piglets weaned		
High-performing farms	56.0 (1.15)ax	54.7 (1.11)ay	45.3 (1.19)az
Intermediate-performing farms	52.1 (0.89)ax	51.2 (0.79)axy	44.7 (0.86)ay
Low-performing farms	46.8 (1.42)bx	46.5 (1.12)bxy	38.7 (1.22)by
	Lifetime non-productive days		
High-performing farms	82.1 (2.87)cx	80.2 (2.82)cy	73.1 (2.93)cz
Intermediate-performing farms	96.2 (2.13)bx	95.5 (1.99)by	90.8 (2.10)bz
Low-performing farms	111.8 (3.25)ax	109.3 (2.80)ay	103.8 (2.94)az
	Annualized lifetime piglets weaned per sow		
High-performing farms	25.8 (0.20)ax	25.8 (0.19)ax	24.5 (0.21)ay
Intermediate-performing farms	23.4 (0.16)bx	23.1 (0.14)by	21.3 (0.15)bz
Low-performing farms	20.9 (0.26)cx	20.6 (0.19)cx	18.3 (0.21)cy

^a-c^Different superscripts within a column represent significant differences in means (*P* <  0.01)

^x-z^Different superscripts within a row represent significant differences in means (P <  0.01)

^1^Means and SE were estimated by mixed models

^2^Groups based on the upper and lower 10th percentiles of piglets born alive in parity 1: High (15 piglets or more); Intermediate (8 to 14 piglets) and Low (7 piglets or fewer) prolific sows

^3^Categorized by farm means of the upper and lower 25th percentiles of annualized lifetime piglets weaned per sow over 6 years: High- (> 24.7 piglets); Intermediate- (24.7 to 21.2 piglets) and Low- (< 21.2 piglets) performing farms

^4^There were no two-way interactions for ages at first-mating, parity at removal or lifetime non-productive days (*P* ≥ 0.05)

There were significant main effects of sow groups on age at first service and the number of parity at removal (*P* <  0.05), but no effect of farm groups (*P* = 0.35 for age at first service; *P* = 0.44 for parity at removal). Furthermore, there were no 2-way interactions for age at first service, the number of parity at removal or lifetime non-productive days (*P* ≥ 0.05). For example, there were no differences between farm groups for age at first service or number of parity at culling, but H-prolific sows had 1.0–3.5 days greater age at first service and 0.5–0.9 higher number of parity at removal than L-prolific sows across the farm groups.

## Discussion

Our study showed that different farm effects could alter sows’ reproductive potential across parities and lifetime performance of sows. Also, our study indicated that farm effects were greater than sow potential on farrowing rates, non-productive sow days and annualized lifetime piglets weaned, but that sow potential had a greater effect than farm effects on lifetime PBA. Additionally, the 6–15% more PBA across sow groups after parity 1 on HP farms than on LP farms indicates that PBA was not only affected by sow potential, but also by farm effects. In particular, L-prolific sows on HP farms had 12% or more PBA and piglets weaned than L-prolific sows on LP farms, suggesting that HP farms are better than LP farms at exploiting the potential of L-prolific sows.

In addition, our study showed that farrowing rates were 7.7% higher on HP farms than on LP farms, but that farrowing rates were only 0.7% higher in H-prolific sows than in L-prolific sows. This result clearly shows that farm effects had at least 10 times greater impact on farrowing rates than sows’ potential. These farm effects probably include better insemination timing, more advanced technologies [9, 10], better care in the breeding phase [18] and a stricter culling policy [19] on HP farms than on LP farms.

The approximately 27–30% fewer lifetime non-productive days across sow groups on the HP farms than on the LP farms indicates that HP farms could decrease non-productive days not just by having sows with better potential, but also by farm effects. High productive farms have shorter re-service intervals than low productive farms [14] that can be achieved through better breeding and culling practices. Additionally, in parities 1 and 6 in our study HP farms had more H-prolific sows and fewer L-prolific sows than LP farms. The result suggests that the HP farms probably had better feeding, better breeding practices, better care for sows at high risk of low productivity and stricter culling guidelines [20, 21] than the LP farms.

Our study also showed a notable decrease in PBA after parity 1 in H-prolific sows, whereas PBA increased after parity 1 in L-prolific sows. There is a hypothetical cascade from follicle development and embryo survival to pregnancy maintenance in sows [22]. Therefore, while H-prolific gilts may have had more potential than L-prolific gilts, for example, more ova, higher embryo survival and higher progesterone concentrations to maintain pregnancy, their ovarian function from ovaries to pregnancy decreased. One possible reason for this decrease in H-prolific sows is that their ovaries and uterus endometrium may not have had enough time to recover from continuous ovulations and farrowing. A decreased farrowing-to-mating interval decreases the total number of piglets born [23] and PBA at subsequent parity. Meanwhile, low prolific gilts may be associated with having litter of origin problems, such as low birth weight [24]. Therefore, our study suggests that differences in farm effects can affect patterns of reproductive performance in both H-prolific and L-prolific sows. Such differences in farm effects will include differences in gilt development, such as diet and boar exposure [25], facilities and workers’ stockmanship [26].

The lack of any association between either the sow groups or farm groups and weaning-to-first-mating intervals in our present study is similar to the findings in a previous study in Japan [3]. This lack of association may be due to the fact, that weaning-to-first-mating interval is highly related to gonadotropin secretion of sows, which in turn is affected by lactation management including feed intake [15, 27]. Also, the three farm groups had similar policy for ages at first-mating of approximately 250 days, and there was no association between the farm groups and age at-first mating.

Finally, there are some limitations that should be noted when interpreting the results of this observational study using herd data. Health status, nutritional programs and genotype were not taken into account in the analyses. Also, our data contained lifetime records from herd-entry to removal, so our data were not all current. However, even with such limitations, this research provides valuable information for pig producers and veterinarians about the impact of sow potential and farm effects on lifetime reproductive performance of sows.

## Conclusions

Farm effects substantially affected reproductive performance across parities and lifetime performance of sows. Using sows with similar potential at parity 1, HP farms exploited lifetime productivity of sows better than on LP farms, especially L-prolific sows. The higher lifetime productivity of sows on HP farms than on LP farms was due to 8% higher farrowing rate, 27–30% fewer non-productive days and 7–11% more PBA during lifetime. Also, in parity 6 there were 15% or more H-prolific sows on HP farm than on LP farms.

## Appendix A

**Table 7:** For high prolific sows^a^, estimates of fixed factors and random effect variance included in the final linear mixed effects models for number of piglets born alive and for piglets weaned

	Piglets born alive		Piglets weaned	
Fixed and random effects^b,c^	Estimate (± SE)	*P*-value	Estimate (± SE)	*P*-value
Intercept	12.31 (0.20)	< 0.01	9.35 (0.15)	< 0.01
Farm groups^d^		< 0.01		< 0.01
High-performing (HP) farms	1.00 (0.22)		1.64 (0.18)	
Intermediate-performing (IP) farms	0.38 (0.22)		0.95 (0.17)	
Parity groups (Py)		< 0.01		< 0.01
1	3.32 (0.20)		0.96 (0.14)	
2	0.01 (0.20)		0.64 (0.15)	
3	0.41 (0.21)		0.61 (0.15)	
4	0.52 (0.21)		0.55 (0.15)	
5	0.21 (0.21)		0.53 (0.16)	
Farm x Py		< 0.01		< 0.01
HP farms x Py 1	- 0.81 (0.21)		- 0.62 (0.15)	
HP farms x Py 2	- 0.25 (0.21)		- 0.25 (0.15)	
HP farms x Py 3	- 0.05 (0.22)		- 0.28 (0.16)	
HP farms x Py 4	- 0.07 (0.22)		- 0.38 (0.16)	
HP farms x Py 5	0.07 (0.22)		- 0.47 (0.16)	
IP farms x Py 1	- 0.24 (0.22)		- 0.36 (0.16)	
IP farms x Py 2	- 0.04 (0.22)		- 0.17 (0.16)	
IP farms x Py 3	0.01 (0.23)		- 0.30 (0.16)	
IP farms x Py 4	- 0.24 (0.23)		- 0.36 (0.17)	
IP farms x Py 5	- 0.11 (0.23)		- 0.37 (0.17)	
Intercept variance at farm level	0.19 (0.04)	–	0.18 (0.03)	–
Intercept variance at sow level	0.12 (0.01)	–	0.07 (0.01)	–
ICC (records within the same farm), %	2.4	–	4.3	–
ICC (records within the same sow), %	4.0	–	6.0	–

^a^High prolific sows are sows farrowed 15 or more piglets born alive at parity 1 (based on the upper 10th percentile of piglets born alive in parity 1)

^b^SE: standard error; ICC: intraclass correlation coefficient

^c^Reference categories were the LP farms and parity 6 sows

^d^Categorized by farm means of the upper and lower 25th percentiles of annualized lifetime piglets weaned per sow: High- (> 24.7 piglets); Intermediate- (24.7 to 21.2 piglets) and Low- (< 21.2 piglets) performing farms

## Appendix B

**Table 8:** For low prolific sows^a^, estimates of fixed factors and random effect variance included in the final linear mixed effects models for number of piglets born alive and for piglets weaned

	Piglets born alive		Piglets weaned	
Fixed and random effects^b,c^	Estimate (± SE)	P-value	Estimate (± SE)	*P*-value
Intercept	11.36 (0.14)	< 0.01	9.66 (0.13)	< 0.01
Farm groups^d^		< 0.01		< 0.01
High-performing (HP) farms	1.34 (0.18)		1.45 (0.18)	
Intermediate-performing (IP) farms	0.52 (0.16)		0.68 (0.16)	
Parity groups (Py)		< 0.01		< 0.01
1	- 6.24 (0.12)		- 1.38 (0.10)	
2	- 1.00 (0.12)		0.18 (0.10)	
3	- 0.18 (0.13)		0.23 (0.10)	
4	- 0.20 (0.13)		0.32 (0.11)	
5	- 0.09 (0.13)		0.25 (0.11)	
Farm x Py		< 0.01		< 0.01
HP farms x Py 1	- 1.38 (0.17)		0.79 (0.13)	
HP farms x Py 2	0.09 (0.17)		0.01 (0.14)	
HP farms x Py 3	- 0.02 (0.17)		- 0.07 (0.14)	
HP farms x Py 4	0.33 (0.18)		- 0.06 (0.14)	
HP farms x Py 5	0.30 (0.17)		- 0.10 (0.15)	
IP farms x Py 1	- 0.26 (0.15)		0.61 (0.12)	
IP farms x Py 2	0.16 (0.15)		- 0.06 (0.12)	
IP farms x Py 3	- 0.04 (0.16)		- 0.06 (0.13)	
IP farms x Py 4	0.16 (0.16)		- 0.26 (0.13)	
IP farms x Py 5	- 0.07 (0.16)		- 0.25 (0.13)	
Intercept variance at farm level	0.15 (0.03)	–	0.23 (0.04)	–
Intercept variance at sow level	0.16 (0.01)	–	0.07 (0.01)	–
ICC (records within the same farm), %	1.9	–	4.5	–
ICC (records within the same sow), %	3.9	–	6.0	–

^a^Low prolific sows are sows farrowed 7 or fewer piglets born alive at parity 1 (based on the lower 10th percentile of piglets born alive in parity 1)

^b^*SE* standard error, *ICC* intraclass correlation coefficient

^c^Reference categories were Low-performing farms and parity 6 sows

^d^Categorized by farm means of the upper and lower 25th percentiles of annualized lifetime piglets weaned per sow: High- (> 24.7 pigs); Intermediate- (24.7 to 21.2 pigs) and Low- (< 21.2 pigs) performing farms

## Appendix C

**Table 9:** Estimates of fixed factors and random effect variance included in the final models^a^ for farrowing rate and for weaning-to-first-mating interval of served females

	Farrowing rate		Weaning-to-first-mating interval	
Fixed and random effects^b,c^	Estimate (± SE)	P-value	Estimate (± SE)	*P*-value
Intercept	1.45 (0.08)	< 0.01	5.67 (0.16)	< 0.01
Farm groups^d^		< 0.01		0.45
High-performing farms	0.63 (0.08)		- 0.28 (0.22)	
Intermediate-performing farms	0.28 (0.07)		- 0.10 (0.19)	
Parity groups (Py)		< 0.01		< 0.01
0	0.18 (0.06)		–	
1	- 0.32 (0.06)		1.65 (0.03)	
2	- 0.05 (0.06)		0.50 (0.03)	
3	0.03 (0.07)		0.37 (0.03)	
4	0.07 (0.07)		0.21 (0.03)	
5	0.03 (0.07)		0.09 (0.03)	
Sow groups^e^		< 0.01		0.58
High-prolific sows	- 0.15 (0.07)		- 0.03 (0.04)	
Intermediate-prolific sows	- 0.03 (0.06)		- 0.03 (0.02)	
Py x Sow groups		< 0.01		–
Py 0 x High-prolific sows	0.20 (0.08)		–	
Py 0 x I-prolific sows	0.20 (0.07)		–	
Py 1 x High-prolific sows	0.39 (0.08)		–	
Py 1 x Intermediate-prolific sows	0.21 (0.07)		–	
Py 2 x High-prolific sows	0.37 (0.09)		–	
Py 2 x Intermediate-prolific sows	0.23 (0.07)		–	
Py 3 x High-prolific sows	0.24 (0.09)		–	
Py 3 x Intermediate-prolific sows	0.11 (0.07)		–	
Py 4 x High-prolific sows	0.14 (0.09)		–	
Py 4 x Intermediate-prolific sows	0.04 (0.07)		–	
Py 5 x High-prolific sows	0.09 (0.09)		–	
Py 5 x Intermediate-prolific sows	0.04 (0.08)		–	
Intercept variance at farm level	0.07 (0.01)	–	0.59 (0.09)	–
Intercept variance at sow level	0.03 (0.01)	–	0.07 (0.01)	–
ICC (records within the same farm), %	2.2	–	2.9	–
ICC (records within the same sow), %	3.2	–	3.2	–

^a^Logistic regression model and linear mixed effects model were used respectively for farrowing rate and weaning-to-first-mating interval

^b^SE: standard error; ICC: intraclass correlation coefficient

^c^Reference categories were the Low-performing farms, parity 6 sows and Low-prolific sows

^d^Categorized by farm means of the upper and lower 25th percentiles of annualized lifetime piglets weaned per sow: High- (> 24.7 piglets); Intermediate- (24.7 to 21.2 piglets) and Low- (< 21.2 piglets) performing farms

^e^Groups based on the upper and lower 10th percentiles of piglets born alive in parity 1: High- (15 piglets or more); Intermediate- (8 to 14 piglets) and Low- (7 piglets or fewer) prolific sows

## Appendix D

**Table 10:** Mean values of reproductive performance between the three farm productivity groups of either high prolific or low prolific sows in consecutive parities^a,b^

		Consecutive parity						
Farm groups^c^	N^d^	0	1	2	3	4	5	6
		High prolific sows^e^						
		Farrowing rate, %						
High-performing farms	6624	91.5 (0.57)	88.4 (0.72)	90.5 (0.64)	90.1 (0.67)	89.6 (0.72)	88.6 (0.80)	86.9 (0.96)
Intermediate-performing farms	3744	87.3 (0.74)	83.6 (0.88)	87.1 (0.79)	85.4 (0.88)	84.5 (0.97)	84.1 (1.05)	83.2 (1.22)
Low-performing farms	784	81.7 (1.65)	79.0 (1.81)	81.6 (1.77)	84.5 (1.71)	84.3 (1.84)	78.5 (2.34)	77.2 (2.84)
		Weaning-to-first-mating interval, days						
High-performing farms	6139	–	7.2 (0.19)	5.9 (0.19)	5.8 (0.19)	5.7 (0.19)	5.5 (0.19)	5.3 (0.20)
Intermediate-performing farms	3447	–	7.2 (0.15)	6.1 (0.16)	6.0 (0.16)	5.9 (0.16)	5.7 (0.17)	5.5 (0.18)
Low-performing farms	715	–	7.3 (0.25)	6.0 (0.26)	6.4 (0.26)	5.7 (0.27)	5.9 (0.29)	5.6 (0.33)
		Low prolific sows^e^						
		Farrowing rate, %						
High-performing farms	3332	90.2 (0.69)	84.5 (0.96)	87.2 (0.90)	88.9 (0.87)	89.9 (0.88)	88.4 (1.04)	87.6 (1.33)
Intermediate-performing farms	4575	87.3 (0.64)	81.1 (0.84)	84.5 (0.78)	85.2 (0.80)	85.0 (0.86)	85.5 (0.92)	83.8 (1.15)
Low-performing farms	2213	83.6 (1.05)	75.3 (1.37)	80.0 (1.28)	80.4 (1.34)	81.7 (1.39)	80.2 (1.58)	83.2 (1.62)
		Weaning-to-first-mating interval, days						
High-performing farms	2869	–	7.0 (0.19)	5.8 (0.19)	5.7 (0.19)	5.6 (0.20)	5.5 (0.21)	5.4 (0.24)
Intermediate-performing farms	3948	–	7.3 (0.14)	6.3 (0.14)	6.1 (0.14)	5.8 (0.15)	5.7 (0.16)	5.6 (0.17)
Low-performing farms	1903	–	7.2 (0.19)	6.2 (0.20)	6.1 (0.20)	5.9 (0.21)	6.1 (0.22)	5.7 (0.24)

^a^Means and SE were estimated by mixed models

^b^Significant differences (e.g. a, b) were not shown in this Table, because there were no 3-way interactions between sow groups, farm groups and parity groups for both farrowing rate and the weaning-to-first-mating interval (*P* ≥ 0.05)

^c^Sow groups based on the upper and lower 10th percentiles of piglets born alive in parity 1: High- (15 piglets or more)- and Low- (7 piglets or fewer) prolific sows

^d^N represents the initial number of sows

^e^Categorized by farm means of the upper and lower 25th percentiles of annualized lifetime piglets weaned per sow: High- (> 24.7 piglets); Intermediate- (24.7 to 21.2 piglets) and Low- (< 21.2 piglets) performing farms

## Abbreviations

HP: High-performing
H-prolific: High prolific
ICC: Intraclass correlation coefficient
IP: Intermediate-performing
I-prolific: Intermediate prolific
LP: Low-performing
L-prolific: Low prolific
PBA: Piglets born alive
